# Evaluation of combination treatment with DS-1205c, an AXL kinase inhibitor, and osimertinib in metastatic or unresectable *EGFR*-mutant non-small cell lung cancer: results from a multicenter, open-label phase 1 study

**DOI:** 10.1007/s10637-023-01341-y

**Published:** 2023-03-09

**Authors:** James Chih-Hsin Yang, Wu-Chou Su, Chao-Hua Chiu, Her-Shyong Shiah, Kang-Yun Lee, Te-Chun Hsia, Makiko Uno, Nigel Crawford, Hiroshi Terakawa, Wen-Chi Chen, Gensuke Takayama, Ching Hsu, Ying Hong, Carline Saintilien, Joseph McGill, Gee-Chen Chang

**Affiliations:** 1 National Taiwan University Cancer Center, No. 57, Ln. 155, Sec. 3, Keelung Rd., Da’an Dist., Taipei City, 106 Taiwan; 2 National Cheng Kung University Hospital, Tainan, Taiwan; 3 Taipei Cancer Center and Taipei Medical University Hospital, Taipei Medical University, Taipei, Taiwan; 4 Graduate Institute of Cancer Biology and Drug Discovery, College of Medical Science and Technology, Taipei Medical University Hospital, Taipei, Taiwan; 5 Taipei Medical University-Shuang Ho Hospital, Taipei, Taiwan; 6 China Medical University Hospital, Taichung, Taiwan; 7 Daiichi Sankyo Co. Ltd., Tokyo, Japan; 8 Daiichi Sankyo Inc., Basking Ridge, NJ USA; 9 Daiichi Sankyo Taiwan Ltd, Taipei, Taiwan; 10 Division of Pulmonary Medicine, Department of Internal Medicine, Chung Shan Medical University Hospital, Taichung, Taiwan

**Keywords:** Oncology, Advanced non-small cell lung cancer, Inoperable non-small cell lung cancer, Epidermal growth factor receptor, DS-1205c, AXL kinase inhibitor

## Abstract

The objective of this study was to evaluate the safety and tolerability of DS-1205c, an oral AXL-receptor inhibitor, in combination with osimertinib in metastatic or unresectable *EFGR*-mutant non-small cell lung cancer (NSCLC) patients who developed disease progression during EGFR tyrosine kinase inhibitor (TKI) treatment. An open-label, non-randomized phase 1 study was conducted in Taiwan, in which 13 patients received DS-1205c monotherapy at a dosage of 200, 400, 800, or 1200 mg twice daily for 7 days, followed by combination treatment with DS-1205c (same doses) plus osimertinib 80 mg once daily in 21-day cycles. Treatment continued until disease progression or other discontinuation criteria were met. At least one treatment-emergent adverse event (TEAE) was reported in all 13 patients treated with DS-1205c plus osimertinib; with ≥ 1 grade 3 TEAE in 6 patients (one of whom also had a grade 4 increased lipase level), and 6 patients having ≥ 1 serious TEAE. Eight patients experienced ≥ 1 treatment-related AE (TRAE). The most common (2 cases each) were anemia, diarrhea, fatigue, increased AST, increased ALT, increased blood creatinine phosphokinase, and increased lipase. All TRAEs were non-serious, with the exception of an overdose of osimertinib in 1 patient. No deaths were reported. Two-thirds of patients achieved stable disease (one-third for > 100 days), but none achieved a complete or partial response. No association between AXL positivity in tumor tissue and clinical efficacy was observed. DS-1205c was well-tolerated with no new safety signals in patients with advanced *EGFR-*mutant NSCLC when administered in combination with the EFGR TKI osimertinib. ClinicalTrials.gov; NCT03255083.

**Supplementary Information:**

The online version contains supplementary material available at 10.1007/s10637-023-01341-y.

## Introduction

Non-small cell lung cancers (NSCLCs) with acquired epidermal growth factor receptor (*EGFR*) mutations that are resistant to EGFR tyrosine kinase inhibitors (TKIs) represent a neglected and difficult-to-treat subtype of lung cancers [1–4]. According to a meta-analysis of 456 studies, *EGFR* mutations are found in 32.3% of NSCLC patients, with higher rates in certain countries (38.4% in China), females (43.7% vs. 24.0% in males), non-smokers (49.3% vs. 21.5% in past/current smokers), and patients with adenocarcinoma (38.0% vs. 11.7%; with vs. non-adenocarcinoma) [5].

EGFR TKIs represent an advance in the first-line treatment of *EGFR*-mutant NSCLC with increased response rates, delayed time to progression, and improved overall survival (OS). According to the National Comprehensive Cancer Network^®^ (NCCN) Clinical Practice Guidelines for NSCLC [6], the preferred first-line option for *EGFR*-mutant NSCLC is osimertinib, a third-generation EGFR TKI. Other possible choices include afatinib, dacomitinib, erlotinib and gefitinib [6]. However, resistance to EGFR TKIs invariably occurs within 1-year [1–4, 7], and when this occurs treatment choices are limited [2, 3, 6].

In this challenging environment, options for the subsequent treatment of *EGFR*-mutant NSCLC will vary depending on the type and extent of disease progression [6] and may include: definitive local therapy (e.g., stereotactic ablative radiotherapy or surgery) for limited lesions; continuation of first-line treatment if it has some benefit (e.g., erlotinib, afatinib, gefitinib, or osimertinib unless the cancer is widespread, and continuing dacomitinib unless the cancer is in the brain/widespread); or switching to a different targeted therapy. In *T790M*-positive NSCLC, the preferred second-line option is osimertinib [3, 6, 8], as it specifically targets the EGFR *T790M* mutation, which accounts for 50–60% of cases that are resistant to first- and second-generation EGFR TKIs [3, 7]. In the phase 3 AURA3 study, osimertinib had significantly greater efficacy than platinum therapy plus pemetrexed in patients with *T790M*-positive advanced NSCLC in whom disease had progressed during first-line EGFR-TKI therapy [9]. In patients with disease progression despite EGFR TKI therapy (including second-line osimertinib), treatment may be switched to afatinib plus cetuximab [6]; and, finally, possibly switching to combination chemotherapy based on the NSCLC cell type (i.e., lung adenocarcinoma or squamous cell carcinoma).

Given the limitations of the current treatments, there is a need for alternative approaches to treat progressive *EGFR*-mutant NSCLC. Currently being explored is inhibition of the expression of AXL tyrosine kinase, a cell-surface transmembrane receptor that plays a key role in signal transduction in both normal and malignant cells [10, 11]. Upregulation of AXL has been observed in a variety of tumors resistant to first-line treatments; including *EFGR*-mutant NSCLC that has progressed on EGFR TKIs, especially in *T790M*-negative populations [10–14]. As abnormal expression and activation of AXL may promote chemotherapy resistance, cancer cell proliferation, invasion, and metastasis, blocking AXL pathways may enhance the sensitivity of cancer cells to cytotoxic agents and help to overcome the problem of resistance to EGFR TKIs [10, 12–15]. Therefore, AXL inhibitors may delay the onset of EGFR TKI resistance in selected NSCLC patients [10, 13–15].

DS-1205c is a novel, specific, small-molecule AXL inhibitor that has been developed as an oral antitumor agent for use in combination with EGFR TKIs. In tumor xenograft models, the addition of DS-1205c restored sensitivity to EGFR TKIs in cells with the *EGFR* exon 19 deletion mutation (*T790M*-negative). Additionally, in cell-based assays, addition of DS-1205b (a sulfate trihydrate of DS-1205a, which is the free form of DS-1205c) with erlotinib or osimertinib prolonged the therapeutic benefit of the EGFR TKIs by inhibiting signaling downstream of EGFR; it also inhibited acquired resistance to osimertinib and erlotinib in an *EGFR*-mutant (exon 19 deletion) NSCLC xenograft mouse model [16]. Based on the anti-tumor activity and favorable toxicity profile of DS-1205c in nonclinical models, the use of DS-1205c in combination with an EGFR TKI was investigated in patients with metastatic or unresectable *EGFR*-mutant (*T790M*-negative) NSCLC in a multicenter, open-label, phase 1 Japanese study. In combination with gefitinib, DS-1205c was safe and well-tolerated [17]. Our phase 1 study, conducted in Taiwan, was designed to evaluate the combination of DS-1205c with osimertinib in metastatic or unresectable *EGFR*-mutant NSCLC. In this report we present the key safety/tolerability and efficacy data from the study; with pharmacokinetic (PK) and biomarker findings presented as Supplementary Materials.

## Methods

This multicenter, open-label, phase 1 dose-escalation study (NCT03255083) assessed the safety, tolerability, and antitumor efficacy of DS-1205c in combination with osimertinib in metastatic or unresectable *EGFR*-mutant NSCLC. Patients either had disease progression (but were *T790M*-negative after being treated with afatinib, erlotinib or gefitinib) or developed disease progression while receiving osimertinib. The study was conducted at seven Taiwanese institutes from April 2019 to September 2020.

### Objectives

The primary objective was to assess the safety and tolerability of DS-1205c in combination with osimertinib in metastatic or unresectable *EGFR*-mutant NSCLC patients who developed disease progression during EGFR TKI treatment.

Secondary objectives included: preliminary evaluation of the antitumor efficacy of DS-1205c plus osimertinib, and characterization of the PK profile of DS-1205a following DS-1205c monotherapy and in combination with osimertinib. Exploratory objectives included identifying biomarkers or levels of AXL-positivity in tumor cells that correlate with the treatment response or toxicity.

### Inclusion and exclusion criteria

The trial included male/female patients aged ≥ 18 years with histologically/cytologically confirmed metastatic or unresectable *EGFR*-mutant NSCLC. Key inclusion criteria were: acquired resistance to an EGFR TKI [18] and clinically defined as a tumor with an *EGFR* mutation associated with EGFR TKI sensitivity [e.g., *G719X*, exon 19 deletion, *L858R, L861Q*], or experienced clinical benefit from continuous EGFR TKI treatment followed by systemic disease progression defined by Response Evaluation Criteria in Solid Tumors version 1.1 (RECIST v1.1 [19]), continuous treatment with erlotinib, gefitinib, afatinib, or osimertinib for ≥ 6 weeks with well-controlled (grade < 3), related toxicities at screening; ≥ 1 measurable tumor based on RECIST v1.1 [19]; absence of the *EGFR T790M* mutation after the completion or the prior treatment (*EGFR* mutation testing not required if previously treated with osimertinib); Eastern Cooperative Oncology Group performance status (ECOG-PS) of 0 or 1 [20]; and adequate bone marrow reserve and organ function. Before the first dose of DS-1205c, patients were required to undergo a treatment washout period of ≥ 10 days if they were receiving medications which prolonged QTc or strongly induced cytochrome P450 3A4, and ≥ 5 days for proton pump inhibitors.

Key exclusion criteria were: evidence of small cell or small cell plus non-small cell histology; previous documentation of *ALK* fusion, *ROS1* fusion, *BRAF* V600E mutations, *RET* rearrangement, *HER2* mutation, or *MET* exon 14 skipping mutation. Further details on inclusion and exclusion criteria are provided in Supplementary Table 1.

Patients provided voluntary written informed consent before participating in any study procedures.

### Treatment

Patients were allocated to receive oral DS-1205c at an assigned dosage of 200, 400, 800, or 1200 mg twice daily (bid; cohorts 1, 2, 3, and 4). The lowest DS-1205c dosage (i.e., 200 mg bid) was based on nonclinical data, while the escalated dosages were guided by a modified continual reassessment method using a Bayesian logistic regression model following the dosage escalation with overdose control principle [21, 22]. For a given dose, the probability of the dose-limiting toxicity (DLT) rate being greater than 33% needed to be less than 0.25. DS-1205c dosages are expressed in terms of the free-base DS-1205a.

Before starting DS-1205c treatment, eligible patients continued to receive their current EGFR TKI (erlotinib, gefitinib, afatinib, or osimertinib) to minimize the likelihood of tumor flare resulting from TKI discontinuation. The last dose of their prior TKI was taken in the morning 1 day before the initial DS-1205c run-in period.

During the run-in period (cycle 0), patients received monotherapy with DS-1205c bid at their assigned dose for 1 week. For cycle 1 and beyond, patients received combination treatment with the assigned dose of DS-1205c bid (every 12 h with a meal) and oral osimertinib 80 mg once daily (co-administered with the morning dose of DS-1205c) [8]. Each combination treatment cycle was 21 days, and these were continued until observed progressive disease (RECIST v1.1 criteria [19]), clinical progression (definite clinical signs of disease progression, but without a recent radiographic assessment meeting the RECIST criteria for progressive disease), or other discontinuation criteria were met.

### Safety outcomes

Safety endpoints included the occurrence of DLTs (any treatment-emergent adverse event [TEAE] which is ≥ Grade 3 and not attributable to the disease process assessed during cycles 0 and 1); AEs including TEAEs, treatment-related AEs (TRAEs), and serious AEs; and changes in standard clinical laboratory parameters, vital signs, electrocardiogram parameters (corrected QT [QTcF] and QRS intervals), physical examination (including ECOG-PS), echocardiogram and multi-gated acquisition scans (e.g., left ventricular ejection fraction [LVEF]), and ophthalmologic findings. Pulmonary assessments were conducted at the discretion of the investigators.

AEs were coded and summarized by system organ class (SOC) and preferred term (PT) using the latest version of the Medical Dictionary for Regulatory Activities (MedDRA) [23]. AEs and laboratory findings were graded according to National Cancer Institute (NCI)-Common Terminology Criteria for Adverse Events (NCI-CTCAE v5.0) [24]. At each level of subject summarization, patients were counted once if they reported ≥ 1 TEAE. TEAEs that occurred more than once for any patient were counted only once in the patient frequencies. TEAEs with different grades for a patient were counted at the highest grade. A TEAE with a missing grade was counted lower than another TEAE with the same PT but having been graded.

### Antitumor efficacy

The primary efficacy endpoint was the ORR, defined as the number of patients with the best objective response of complete response (CR) or partial response (PR), divided by the total number of patients analyzed in the same treatment group. Response was determined by investigator assessment and based on RECIST v1.1. Additional efficacy endpoints included disease control rate (sum of CR, PR, and stable disease rates), PFS, OS, and percentage change in the size of the target lesion. Starting from Day 1 of Cycle 1, tumors were assessed every 6 weeks for the first 24 weeks (i.e., first 8 cycles), then every 12 weeks thereafter (i.e., every 4 cycles).

### Statistical analysis

Analyses of safety, efficacy, pharmacokinetics, and biomarkers were performed on the full patient set. Safety and efficacy data are presented as descriptive summaries, with continuous data presented as means with standard deviation, or medians with range, and categorical data presented as count and percentage. Kaplan-Meier analyses were used to estimate the duration of response, PFS, and OS.

### Ethical approval

This study was performed in accordance with the ethical principles of the Declaration of Helsinki, and the International Council for Harmonization Consolidated Guideline for Good Clinical Practice, and other applicable regulatory requirements. It was approved by the appropriate local Independent Ethics Committees or Institutional Review Boards.

## Results

**Fig. 1:**
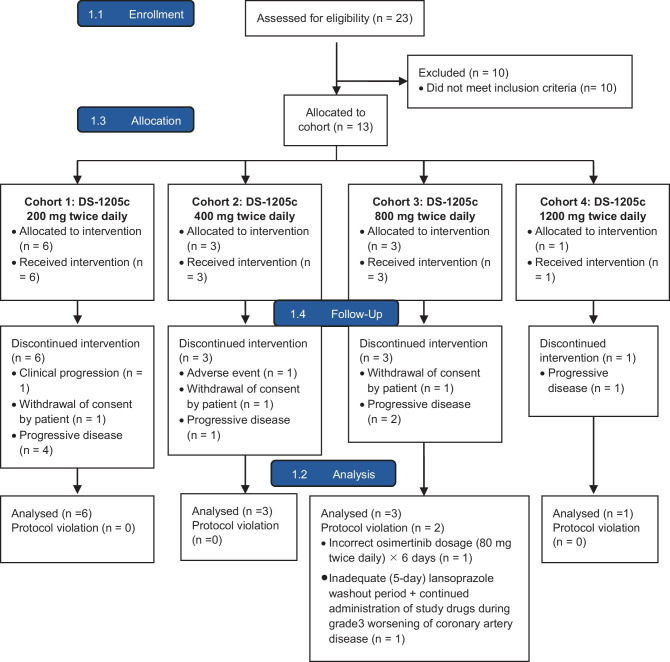
CONSORT patient flow diagram

A total of 23 patients were screened for the study and 13 patients from 6 institutes in Taiwan participated (Fig. 1). Enrollment was terminated after one patient was enrolled in cohort 4 (i.e., the highest dosage of DS-1205c 1200 mg bid), due to changes in the sponsor’s strategic priorities. Termination of enrollment was not because of safety concerns.

All 13 enrolled patients (n = 6, 3, 3, and 1 in cohorts 1, 2, 3, and 4, respectively) were included in the “all analysis set” (Fig. 1). Tumor samples from 11 patients underwent AXL immunohistochemistry evaluation; tumor tissue was insufficient to evaluate by immunohistochemistry for the remaining 2 patients (1 each in cohorts 2 and 3).

### Baseline patient characteristics

At baseline, the median patient age was 64 (range, 45–88) years. All patients were Asian with 10 females (76.9%) and 3 males (23.1%). Seven patients (53.8%) had an ECOG-PS of 0, and the remaining patients had a score of 1. Patients had received a median of 3 (range, 1–11) prior anticancer regimens. All patients had received EGFR TKI treatment immediately before study enrollment, including 8 (61.5%) osimertinib; 3 (23.1%) erlotinib, and 2 (15.4%) afatinib. Further details, including baseline characteristics in each cohort, are presented in Supplementary Table 2.

### Extent of exposure to DS-1205c

Overall, the median duration of DS-1205c treatment was 90 (range, 28–281) days, the median number of treatment cycles was 5 (range, 2–14), and the median relative DS-1205c dose intensity was 99.5% (range, 84.5–100%). Further details of exposure to DS-1205c, including by cohort, are provided in Table 1.

**Table 1:** Exposure to DS-1205c (safety analysis set)

	**Cohort: DS-1205c twice-daily dosage**				
**Parameter**	All: 200–1200 mg (n = 13)	1: 200 mg (n = 6)	2: 400 mg (n = 3)	3: 800 mg (n = 3)	4: 1200 mg (n = 1)
**Exposure to DS-1205c, median (range)**					
Treatment duration, days	90 (28–281)	50 (28–260)	90 (41–281)	154 (90–154)	91
Total amount of drug taken, g	71.6 (9.8–246.4)	20.0 (9.8–104.0)	71.6 (29.6–214.8)	233.6 (144.0–246.4)	218.4
Actual dose intensity, mg/day	722.0 (338–2400)	397.3 (338–400)	764.4 (722–796)	1600.0 (1517–1600)	2400.0
Relative dose intensity, %	99.5(84.5–100.0)	99.4 (84.5–100.0)	95.6 (90.3–99.5)	100.0 (94.8–100.0)	100.0
Total no. of cycles initiated	5 (2–14)	3 (2–13)	5 (4–14)	8 (5–8)	5
**Length of completed treatment**					
≤ 3 months, n	8	4	2	1	1
> 3 to ≤ 6 months, n	3	1	0	2	0
> 6 to ≤ 9 months, n	1	1	0	0	0
> 9 to ≤ 12 months, n	1	0	1	0	0
**Dose reduction or interruption (any reason)**					
Dose reduction, n	0	0	0	0	0
Dose interruption, n	5	2	2	1	0

### Tolerability and safety

During the DLT-evaluation period (Cycle 0, Day 1 to Cycle 1, Day 21), DLT occurred in 1 patient (grade 3 pneumonia and increased ALT levels) at the beginning of Cycle 1 with DS-1205c 200 mg (cohort 1). No DLTs occurred in patients receiving DS-1205c at the higher dosages of 400–1200 mg bid.

During the treatment period no trends in the profile of TEAEs or TRAEs were observed in any of the DS-1205c dosage cohorts. TEAEs with the same PT that occurred in 2 or more patients, other TEAEs with an NCI-CTCAE (v5.0) grade ≥ 3, or serious TEAEs that occurred in ≥ 1 patient, are listed in Table 2. At least one TEAE was reported in all 13 patients; with ≥ 1 NCI-CTCAE v5.0 grade 3 TEAE in 6 patients, one of whom also had a grade 4 TEAE (increased lipase), and 6 patients having ≥ 1 serious TEAE (Table 2).

**Table 2:** Treatment-emergent adverse events in patients receiving twice-daily DS-1205c (safety analysis set)

System organ class	TEAE (preferred term)	Cohort: DS-1205c twice-daily dosage				
		All: 200–1200 mg (n = 13)	1: 200 mg (n = 6)	2: 400 mg (n = 3)	3: 800 mg (n = 3)	4: 1200 mg (n = 1)
**TEAEs reported in > 1 patient, n**						
Infections and infections	URTI	3	2	0	1	0
	UTI	3	2	1	0	0
	Pneumonia	2	1^a^^,b^	0	0	1^a^
Blood and lymphatic system disorders	Anemia	3	0	1^b^^,c^	1^c^	1
Psychiatric disorders	Insomnia	4	1	2	1	0
Nervous system disorders	Dizziness	2	1	1	0	0
	Headache	2	1	0	1	0
Respiratory, thoracic, and mediastinal disorders	Cough	2	1	1	0	0
	Dyspnea	2	1	1	0	0
	Hemoptysis	2	1	1	0	0
Gastrointestinal disorders	Vomiting	4	3	1	0	0
	Diarrhea	3	0	1	2	0
	Constipation	2	2	0	0	0
Skin and subcutaneous tissue disorders	Rash	2	1	1	0	0
General disorders and administration-site conditions	Fatigue	3	1	2	0	0
	Pyrexia	2	0	2	0	0
Investigations	ALT ↑	3	1^a^^,b^	0	1	1
	AST ↑	3	1^b^^,c^	0	1	1
	Blood CPK ↑	3	0	1	1	1
	Blood creatinine ↑	2	0	1	1	0
	Ejection fraction ↓	2	0	1	1^c^	0
	Lipase ↑	2	0	0	1^d^	1
Injury, poisoning, and procedural complications	Overdose (of osimertinib)	2	0	0	2^e^	0
**Serious and/or grade ≥ 3 TEAEs reported in 1 patient (not reported above), n**						
Neoplasms benign, malignant, and unspecified	Metastases to meninges	1	1^f^	0	0	0
Renal and urinary disorders	Hematuria	1	1^b^^,c^	0	0	0
Cardiac disorders	Cardiac failure	1	0	0	1^a^	0
	Worsening CAD	1	0	0	1^a^^,b^	0
Vascular disorders	Worsening hypertension	1	0	1^b^^,c^	0	
Respiratory, thoracic, and mediastinal disorders	Pneumonia aspiration	1	0	1^a^^,b^	0	0
Gastrointestinal disorders	Dysphagia	1	0	1^b^^,g^	0	0
	Pancreatitis	1	0	0	1^a^	0
Investigations	AST ↑	1	1^a^	0	0	0
**Summary of TEAEs, n**						
Any TEAE	13	6	3	3	1	
Any serious TEAE	6	2	1	2	1	
Any TEAE grade ≥ 3	6	3	1	1	1	
Any TEAE leading to dose interruption	4	2	1	1	0	

TEAEs were coded using Medical Dictionary for Regulatory Activities (MedDRA) version 19 and graded using National Cancer Institutes-Common Terminology Criteria for Adverse Events (NCI-CTCAE) version 5.0

Unless other indicated, TEAEs were non-serious, of NCI-CTCAE grade ≤ 2, were not associated with dose interruption, reduction, or discontinuation, and were resolving/resolved

*ALT* alanine aminotransferase, *AST* aspartate aminotransferase, *CAD* coronary artery disease, *CPK* creatine phosphokinase, *TEAE* treatment-emergent adverse event (adverse event that occurred or worsened in severity after study drug initiation until 30 days after the last dose), *URTI* upper respiratory tract infection, *UTI* urinary tract infection, *↑* increased, *↓* decreased

^a^Serious, grade 3 event

^b^Event led to dose interruption

^c^Non-serious grade 3 event

^d^Non-serious grade 4 event

^e^Serious events; one grade 1 and one grade 2 event

^f^Serious, grade 3 event, with a fatal outcome

^g^Serious, grade 3 event, with outcome ‘not recovered/not resolved’

Table 3 lists TRAEs reported in one or more patients by PT and SOC. Eight patients experienced ≥ 1 TRAE. The most common TRAEs (2 cases each) were anemia, diarrhea, fatigue, increased AST, increased ALT, increased blood creatinine phosphokinase, and increased lipase. All TRAEs were grade 2 or less, with the exception of a grade 4 lipase increase in 1 patient in cohort 3, and a grade 3 hematuria in 1 patient in cohort 1; all TRAEs were non-serious, with the exception of an overdose of osimertinib in 1 patient in cohort 3 (Table 3). No deaths were associated with the investigated medication.

**Table 3:** Treatment-related adverse events in patients receiving twice-daily DS-1205c (safety analysis set)

		Cohort: DS-1205c twice-daily dosage				
System organ class	TRAE (preferred term)	All: 200–1200 mg (n = 13)	1: 200 mg (n = 6)	2: 400 mg (n = 3)	3: 800 mg (n = 3)	4: 1200 mg (n = 1)
**TRAEs reported in ≥ 1 patient, n**						
Infections and infections	Paronychia	1	1	0	0	0
Blood and lymphatic system disorders	Anemia	2	0	0	1	1
Endocrine disorders	Hyperthyroidism	1	0	0	1	0
Metabolism and nutrition disorders	Hypoalbuminemia	1	0	0	1	0
Nervous system disorders	Dizziness	1	1	0	0	0
	Diarrhea	2	0	0	2	0
Gastrointestinal disorders	Mucosal inflammation	1	0	0	1	0
	Mucositis oral (stomatitis)	1	0	0	1	0
	Vomiting	1	0	1	0	0
Skin and subcutaneous tissue disorders	Rash	1	1	0	0	0
	Rash maculo-papular	1	1	0	0	0
Renal and urinary disorders	Hematuria	1	1^a^	0	0	0
General disorders and administration-site conditions	Fatigue	2	1	1	0	0
Injury, poisoning, and procedural complications	Overdose (of osimertinib)	1	0	0	1^b^	0
	ALT ↑	2	0	0	1	1
	AST ↑	2	0	1	0	1
	Blood CPK ↑	2	0	1	0	1
Investigations	Lipase ↑	2	0	0	1^c^	1
	Blood bilirubin ↑	1	0	0	1	0
	Platelet count ↓	1	0	0	1	0
	White blood cell count ↓	1	0	0	1	0
	Ejection fraction ↓	1	0	1	0	0
**Summary of TRAEs, n**^**d**^						
Any TRAE		8	3	2	2	1
Any serious TRAE		1	0	0	1	0
Any TRAE grade ≥ 3		2	1	0	1	0
Any TRAE leading to dose interruption		1	1	0	0	0

TRAEs were coded using Medical Dictionary for Regulatory Activities (MedDRA) version 19, and graded using National Cancer Institutes-Common Terminology Criteria for Adverse Events (NCI-CTCAE) version 5.0

Unless otherwise indicated, TRAEs were non-serious, of NCI-CTCAE grade ≤ 2, were not associated with dose interruption, reduction, or discontinuation, and were resolving/resolved

*ALT* alanine aminotransferase, *AST* aspartate aminotransferase, *CPK* creatine phosphokinase, *TRAE* treatment-emergent adverse event (occurred or worsened in severity after study drug initiation until 30 days after the last dose) considered to be related to the study drug, *↑* increased, *↓* decreased

^a^Non-serious grade 3 event; led to dose interruption

^b^Serious grade ≤ 2 event

^c^Non-serious Grade 4 event

^d^Individuals may have had ≥ 1 TRAE

Overall, dose interruption due to TEAEs was required in 4 of 13 patients, including 2 patients in cohort 1 (elevated ALT, AST and subsequent pneumonia in one patient, and hematuria in the other), 1 in cohort 2 (anemia, worsened hypertension, dysphagia, and pneumonia aspiration), and 1 in cohort 3 (worsened coronary artery disease). Of these, hematuria was the only TRAE that led to dose interruption (Table 3). No TEAEs or TRAEs led to study discontinuation or dosage reduction.

Cardiac TEAEs of special interest were reported in 2 patients: 1 in cohort 3 with a grade 3 decrease in LVEF and grade 3 cardiac failure, and 1 in cohort 2 with a grade 2 decrease in LVEF (returned to normal by the final end-of-treatment visit). No clinically significant findings related to cardiac function were noted during the initial 7-day monotherapy period. Allergic reactions were experienced by 5 patients; none worse than grade 2. These reactions included two reports of dyspnea (cohorts 1 and 2), two reports of rash (cohorts 1 and 2), one report of hypotension (cohort 3), one report of pruritus, skin exfoliation, and wheezing each in cohort 2.

Alterations in clinical laboratory parameters, including transaminase levels, were mild, and resolved without the need for treatment changes. No relationship between variations in transaminase levels and DS-1205c dosages was observed.

No clinically relevant changes from baseline were seen in vital signs or ocular toxicity events, and no pulmonary assessments (e.g., for interstitial lung disease) were required. ECOG-PS remained stable at 0 or 1 throughout the study, except in one patient in cohort 2, in whom it worsened to 2.

### Treatment response

Table 4 provides a summary of the overall antitumor efficacy of DS-1205c in combination with osimertinib during the trial, and in the four dosage cohorts separately. For all patients (n = 13), stable disease was achieved in 9 (69.2%), progressive disease in 3 (23.1%) and one patient was regarded as unevaluable. Three patients had stable disease for > 100 days; however, no patients achieved confirmed CR or PR. Based on the best overall response, the disease control rate (DCR) was 69.2% (95% CI 39–91).

**Table 4:** Treatment response and change in tumor size (full analysis set)

Parameter	Cohort: DS-1205c twice-daily dosage				
	All: 200–1200 mg (n = 13)	1: 200 mg (n = 6)	2: 400 mg (n = 3)	3: 800 mg (n = 3)	4: 1200 mg (n = 1)
**Best overall response by investigator assessment**					
CR or PR	0	0	0	0	0
Stable disease, n	9	2	3	3	1
Progressive disease, n	3	3			
Not evaluable, n	1	1			
**Disease control**^a^ **by investigator assessment**					
Disease control, n (%; 95% CI^b^)	9 (69.2; 39–91)	2 (33.3; 4.3–78)	3 (100; 29–100)	3 (100; 29–100)	1 (100; 2.5–100)
**PFS events by investigator assessment**					
Progressive disease, n	9	5	1	2	1
Censored, n	4	1	2	1	
Median PFS, weeks (95% CI^c^)	12.4 (6.9–36.6)	7.1 (5.4–36.6)	NE (12.3–NE)	22.0 (12.1–22.0)	12.4 (NE–NE)
**OS events**					
Death	6	4	1	1	0
Censored, n	7	2	2	2	1
Median OS, weeks (95% CI^c^)	41.9 (32–NE)	46.9 (34–NE)	NE (14–NE)	NE (32–NE)	NE
**Best (minimum)** Δ** in SoD of measurable tumors**					
Δ in SoD ≤ 0.0%, n	4	3		1	
Δ in SoD > 0.0%, n	9	3	3	2	1
Δ in SoD by individual cohort member, %	Range: -15.2 to 81.3	-15.2, -4.3, 0, 6.3, 14.3, 16.7, 81.3	2.6, 4.2, 11.1	-1.0, 8.3, 14.3	7.7

*CI* confidence interval, *CR* complete response, *NE* not evaluable, *OS* overall survival, PFS progression-free survival, *PR* partial response, *SoD* sum of diameters, *Δ* change from baseline

^a^Defined as a CR, PR, or stable disease for ≥ 5 weeks from the date of administration of the first dose of DS-1205c

^b^Based on the exact (Clopper-Pearson) binominal distribution

^c^Computed using the Brookmeyer-Crowley method

Of the 9 patients who achieved a best overall response of stable disease, 5 had received osimertinib as their most recent TKI prior to the start of the study. Two patients with stable disease (one each in cohort 1 and 2) continued study treatment for > 36 weeks without disease progression. One, in cohort 1, had non-targeted brain metastasis that was undetectable on imaging after starting treatment.

Median PFS was 12.4 weeks (95% CI 6.9–36.6) and median OS was 41.9 weeks (95% CI 31.7–not evaluable) weeks (Table 4). Kaplan-Meier plots of PFS and OS are shown in Supplementary Fig. 1.

Most tumors increased in size from baseline (an increase of 3–81% in 9 patients); there was no change in 1 patient, and a decrease of 1–15% in 3 patients (Table 4). In the 8 patients who received osimertinib immediately before study enrollment, tumor shrinkage of 4% and 15% was observed in 2 patients, tumor size increases of 3–16% occurred in 5 patients, and 1 patient had an increase of 81% in tumor size.

## Discussion

This open-label, phase 1 study provided novel findings regarding the safety and tumor response of DS-1205c, a kinase inhibitor that selectively targets AXL, in combination with osimertinib in 13 patients with metastatic or unresectable *EGFR*-mutant NSCLC. At dosages of DS-1205c 200–1200 mg bid, all patients completed at least the first 21-day cycle of combination treatment, with a median treatment duration of 90 days.

Overall, a small number of TRAEs were reported and assessment of the relationship between DS-1205c dosage and any TRAE was not possible. Although at least one TEAE or TRAE occurred in 100% and 61.5% of patients, respectively, no TRAEs were serious, and grade ≥ 3 TRAEs were limited to one case of non-serious grade 3 hematuria in a patient receiving 200 mg bid, and one case of a non-serious grade 4 increase in lipase in a patient receiving DS-1205c 800 mg bid (the only clinically significant change from in a laboratory value). No patients had a TRAE that required treatment discontinuation, and only 1 patient had a TRAE requiring study treatment interruption. A patient receiving DS-1205c (200 mg bid) developed a DLTs (grade 3 pneumonia and increased ALT) and no DLTs occurred at higher dosages. No treatment-related safety signals associated with vital signs, physical examination findings, electrocardiogram, or ECOG-PS were observed. Moreover, no clinically significant findings on cardiac function were shown during the initial 7-day DS-1250c monotherapy run-in period. The safety profile of DS-1205c in combination with osimertinib was consistent with that of DS-1205c in combination with gefitinib reported previously [17], and this favorable safety profile reflects the high selectivity of DS-1205c for AXL [16].

Stable disease was the best overall response with DS-1205c and was achieved by the majority (69.2%) of patients. As no patients achieved a CR or PR, the DCR was also 69.2%. The response did not appear to be related to the dosage of DS-1205c, as stable disease was achieved by ≥ 1 patient in each dosage cohort. Stable disease beyond 100 days was experienced by 3 patients, and beyond 200 days by 2 patients (1 each receiving twice-daily DS-1205c 200 and 400 mg). In the 9 patients that achieved SD, most (n = 6; 66.7%) had received osimertinib as their most recent TKI before the start of the study, 2 patients (both of whom had osimertinib as their most recent TKI) appeared to have tumor shrinkage, and PFS ranged from 6.9 weeks to longer than 36 weeks.

The PK analysis demonstrated that exposure to DS-1205a tended to increase in a less than dose-proportional manner, the PK profile of DS-1205c did not appear to be affected by the coadministration of osimertinib, and the pharmacokinetics of osimertinib and its metabolites AZ5104 and AZ7550 were not affected by DS-1205c (see Supplementary File 1). Treatment with DS-1205c increased plasma levels of the biomarker, soluble AXL, but not IL-8 and osteopontin. This is similar to results from a previous study, in which 9 of 19 evaluable tumors were AXL-positive after EGFR-TKI treatment [25].

The results of our study are limited by its small sample size, which hindered our ability to determine if there was a correlation between exploratory outcomes and clinical efficacy. The heterogeneity in the mutation status of patients, as well as in the prior use of different EGFR TKIs may have influenced the results obtained with DS-1205c plus osimertinib. During preclinical research with DS-1205b (the sulfate trihydrate of DS-1205a [the free form of DS-1205c]), high expression of AXL was observed in tumors that were resistant to long-term administration of osimertinib, while the combination of DS-1205b and osimertinib strongly suppressed tumor growth [16]. In an HCC827 EGFR-mutant NSCLC xenograft mouse model, combination treatment with DS-1205b and erlotinib significantly delayed the onset of tumor resistance compared to erlotinib monotherapy, and DS-1205b restored the antitumor activity of erlotinib in erlotinib-resistant tumors [16]. No obvious tumor shrinkage was seen in the current clinical study. The involvement of AXL inhibition may explain this because various resistance factors are expected to occur as a result of previous EGFR TKI treatment. Therefore, the involvement of AXL may become more apparent when used in combination with earlier TKI treatment.

## Conclusion

This phase 1 study demonstrated that DS-1205c was well-tolerated in patients with advanced *EGFR-*mutant NSCLC when administered in combination with osimertinib. No new safety/tolerability signals were identified. Overall, two-thirds of patients achieved stable disease, with approximately one-third achieving stable disease beyond 100 days. However, no patients achieved a CR or PR.

## Supplementary Information

Below is the link to the electronic supplementary material.

Supplementary file1 (DOCX 96 KB)

## Data Availability

The data that support the findings of this study are available from the corresponding author upon reasonable request.

## List of Uncommon Abbreviations

ALK: Anaplastic lymphoma kinase
BRAF: Proto-oncogene B-Raf
DLT: Dose-limiting toxicity
EGFR: Epidermal growth factor receptor
HER2: Human epidermal growth factor receptor 2
MET: Mesenchymal-epithelial transition
PT: Preferred term
RET: Rearranged during transfection
ROS1: ROS proto-oncogene 1
TKI: Tyrosine kinase inhibitor
