# Supplementary material for: Evaluation of combination treatment with DS-1205c, an AXL kinase inhibitor, and osimertinib in metastatic or unresectable EGFR-mutant non-small cell lung cancer: results from a multicenter, open-label phase 1 study
Source: Invest New Drugs. 2023 Mar 9;41(2):306–16. doi: 10.1007/s10637-023-01341-y (PMC10140009; doi:10.1007/s10637-023-01341-y)
Supplement: Supplementary file 1 — Supplementary file1 (DOCX 96 KB) [file 10637_2023_1341_MOESM1_ESM.docx]

**Supplementary Materials**

**Supplementary File 1. Pharmacokinetics**

**Pharmacokinetic profile methods**

Non-compartmental analysis was used to calculate the pharmacokinetics of DS-1205a and osimertinib in plasma. To assess the pharmacokinetics of DS-1205a, the maximum observed DS-1205a concentration (C_max_), the actual sampling time to reach C_max_ (T_max_), minimum DS-1205a concentrations prior to the beginning, or at the end, of a dosing interval (C_trough_), area under the concentration-time curve (AUC) from time 0 to the last quantifiable time point (AUC_0–last_), AUC during a dosing interval (AUCτ), and other appropriate parameters, were measured on Days 1 and 7 of Cycles 0, 1, and 2. The lower limit of quantification for DS-1205a was 5 ng/mL. For osimertinib and its two active metabolites (AZ5104 and AZ7550), C_max_, T_max_, AUC_0–last_, and AUCτ were measured on Day 1 of Cycles 1 and 2. The lower limit of quantification for osimertinib and its two metabolites was 1.0 ng/mL.

For PK parameters, descriptive summary statistics were calculated by dose level/cycle/study day, and a power model was used to evaluate the relationship between C_max_ and AUC.

**Pharmacokinetic profile results**

Table A provides descriptive PK data for DS-1205a, the free form of DS-1205c, on Cycle 0, Day 1 (start of DS-1205c run-in period), Cycle 0, Day 7 (end of DS-1205c run-in), and Cycle 2, Day 1 (after 21 days of DS-1205c plus once-daily osimertinib 80 mg).

Mean exposure to DS-1205a increased as the dosage of DS-1205c increased. The power model estimated that the 90% CI of the slopes for C_max_ and AUC_0–last_ were less than 1 (Table B), suggesting DS-1205a exposure increased in a less than dose-proportional manner.

For each DS-1205c dosage, mean C_max_, AUC_0–last_, and AUC_τ_ values on Cycle 0, Day 7 were similar to those on Cycle 2, Day 1, and all were higher than those observed on corresponding values on Cycle 0, Day 1 (Table 4). The C_trough_ values (Supplementary File 1. Table A) indicated that there was an accumulation of plasma DS-1205a that increased with the increasing dosage of DS-1205c, and it appeared to reach steady-state after about 7 days of twice-daily oral administration. Using AUC_0–last_ values on Cycle 0, Day 7, and Cycle 0, Day 1, the accumulation ratio of DS-1205a was calculated to be ≈2.5. Across all dosages and cycles, DS-1205a had a median T_max_ of 2.1–6.2 hours, and mean t_1/2_ of 3.3–11.7 hours (Supplementary File 1. Table A).

Mean exposure to DS-1205a (C_max_ and AUC_0-ast_) prior to osimertinib administration (Cycle 0, Day 7) was comparable to that after 21 days of osimertinib administration (Cycle 2, Day 1) [Table A]. Likewise, mean values of osimertinib exposure and t_1/2_ were consistent across all DS-1205c dosages.

Following administration of osimertinib on Day 1 of Cycle 2, mean concentrations of both osimertinib metabolites (AZ5104 and AZ7550) were usually 35–40 ng/mL across the 1–12 hours post-administration period. As the PK parameters for AZ5104 and AZ7550 were similar across all DS-1205c dosage cohorts, pooled results are provided (Table C). The ratio of metabolite to parent osimertinib (calculated using molecular weight-adjusted AUC_0–last_) was 13.7% and 13.6% for AZ5104 and AZ7550, respectively.

In conclusion, the PK analysis demonstrated that exposure to DS-1205a tended to increase in a less than dose-proportional manner. Mean plasma C_trough_ levels of DS-1205a appeared to reach near-plateau concentrations after about 7 days of twice-daily administration. The PK profile of DS-1205c did not appear to be affected by the coadministration of osimertinib 80 mg/day, as the values prior to osimertinib administration were similar to those after 21 days of osimertinib administration. Likewise, the pharmacokinetics of once-daily osimertinib 80 mg and its metabolites AZ5104 and AZ7550 did not appear to be affected by the coadministration of DS-1205c, as values were similar across all DS-1205c cohorts. Based on the fact that DS-1205c doses of 400 mg and above in the current study produced exposure higher than IC_50_ values for AXL phosphorylation in NIH3T3-AXL cell lines [16], it appears that the drug concentration reached a biologically effective dose.

**Supplementary File 1: Table A.** Pharmacokinetic parameters of DS-1205a when administered alone during the initial DS-1205c 1-week run-in period (Cycle 0, Day 1, and Cycle 0, Day 7) and in combination with osimertinib 80 mg/day (Cycle 2, Day 1) [full analysis set]

| **Cohort**  Cycle (C), Day (D) | **Pharmacokinetics of DS-1205a** | | | | | |
| --- | --- | --- | --- | --- | --- | --- |
|  | C_max_ (ng/mL), mean ± SD | AUC_0–last_ (h∙ng/mL), mean ± SD | AUC_τ_ (h∙ng/mL), mean ± SD | T_max_ (h), median (range) | C_trough_ (ng/mL), mean ± SD | t_½_ (h), mean ± SD |
| **1: DS-1205c 200 mg twice daily (n = 6, unless otherwise indicated)** | | | | | | |
| C0, D1 | 292 ± 94.8 | 1916 ± 530 | 1975 ± 563 | 4.1 (2.0–6.0) | NA | 6.98^a^ ± 1.89 |
| C0, D7 | 561 ± 192 | 4193 ± 1514 | 4818 ± 1772 | 4.0 (2.0–4.2) | 376 ± 143 | 11.6^b^ ± 6.7 |
| C2, D1 (+ OSI) | 544^a^ ± 176 | 4445^a^ ± 1389 | 4596^a^ ± 1488 | 4.1^a^ (4.0–4.2) | 359^a^ ± 137 | 9.8^c^ ± 3.3 |
| **2: DS-1205c 400 mg twice daily (n = 3, unless otherwise indicated)** | | | | | | |
| C0, D1 | 413 ± 94.0 | 2493 ± 277 | 2557 ± 257 | 4.0 (3.9–4.0) | NA | 5.3 ± 1.0 |
| C0, D7 | 827 ± 299 | 5716 ± 1931 | 6496 ± 2208 | 4.0 (2.1–4.0) | 460 ± 219 | 7.1^d^ ± 1.0 |
| C2, D1 (+ OSI) | 845 ± 328 | 6852 ± 2716 | 7117 ± 2883 | 4.0 (2.0–4.1) | 576 ± 275 | 7.9 ± 0.8 |
| **3: DS-1205c 800 mg twice daily (n = 3, unless otherwise indicated)** | | | | | | |
| C0, D1 | 499 ± 222 | 2891 ± 1398 | 3002 ± 1472 | 3.9 (2.0–4.0) | NA | 4.7 ± 0.6 |
| C0, D7 | 1041 ± 460 | 7385 ± 4562 | 6972^e^ | 4.0 (3.9–5.9) | 688 ± 631 | 6.9^e^ |
| C2, D1 (+ OSI) | 921 ± 260 | 7220 ± 2629 | 7640 ± 2918 | 2.1 (0–4.0) | 722 ± 406 | 7.0 ± 1.3 |
| **4: DS-1205c 1200 mg twice daily (n = 1)** | | | | | | |
| C0, D1 | 703 | 5050 | 5255 | 6.2 | NA | 3.3 |
| C0, D7 | 1071 | 8602 | 10,300 | 4.0 | 816 | NC |
| C2, D1 (+ OSI) | 1150 | 9495 | 10,000 | 3.9 | 903 | 11.7 |

AUC, area under the concentration-time curve; AUC_0–last_; AUC from time 0 until the last quantifiable time point; AUC_τ,_, AUC during a dosing interval; C_max_, maximum plasma concentration; C_trough_, trough concentration (the pre-dose observed concentration immediately before the next dose or at the end of a dosing interval); NA, not applicable; NC, not calculable; OSI, once-daily osimertinib 80 mg; SD, standard deviation, T_max_, time of maximum concentration; t_½,_ half-life.

^a^ Data from 5 patients was used to determine the pharmacokinetic parameter.

^B^ Data from 4 patients was used to determine the pharmacokinetic parameter.

^c^ Data from 3 patients was used to determine the pharmacokinetic parameter.

^d^ Data from 2 patients was used to determine the pharmacokinetic parameter.

^e^ Data available for only 1 patient for the pharmacokinetic parameter.

**Supplementary File 1: Table B.** Summary of the results of the power model of the pharmacokinetic parameters of twice-daily DS-1205c 200, 400, 800, or 1200 mg

| **Time point** | **Slope (90% CI)** | |
| --- | --- | --- |
|  | **C_max_** | **AUC_0–last_** |
| Cycle 0, Day 1 | 0.431 (0.179–0.682) | 0.388 (0.144–0.631) |
| Cycle 0, Day 7 | 0.416 (0.152–0.679) | 0.385 (0.082–0.687) |
| Cycle 2, Day 1 | 0.413 (0.151–0.674) | 0.394 (0.116–0.672) |

Power model included available data from patients in any of the four DS-1205c dosage cohorts.

Power model: Y = β0 + β1 🞨 X, where: Y = logarithm of the pharmacokinetic parameters C_max_ (ng/mL) or AUC_0–last_ (h∙ng/mL); β0 = intercept; β1 = slope of the power model; and X = logarithm of the DS-1205c dosage.

AUC_0–last_, area under the concentration-time curve from time 0 until the last quantifiable time point; CI, confidence interval; C_max_, maximum plasma concentration.

**Supplementary File 1: Table C.** Pharmacokinetic parameters of oral osimertinib 80 mg once daily and its metabolites AZ5104 and AZ7550 on Cycle 2, Day 1 of treatment with oral DS-1205c (200, 400, 800 or 1200 mg twice daily)

| Cohort: DS-1205c twice-daily dosage | **Pharmacokinetic parameters** | | | | |
| --- | --- | --- | --- | --- | --- |
|  | C_max_ (ng/mL), mean ± SD | AUC_0–last_ (h∙ng/mL), mean ± SD | AUC_τ_ (h∙ng/mL), mean ± SD | T_max_ (h), median (range) | t_½_ (h), mean ± SD |
| **Osimertinib** | | | | | |
| All: 200–1200 mg (n = 12^a^) | 341 ± 117 | 6753 ± 2379 | 7017^b^ ± 2381 | 6.07 (2.02–12.2) | 53.8^c^ ± 18.8 |
| 1: 200 mg (n = 5^a^) | 376 ± 162 | 7708 ± 3305 | 8455^d^ ±3232 | 10 (3.95–12.2) | NC |
| 2: 400 mg (n = 3^a^) | 308 ± 46.5 | 6206 ± 1131 | 6200 ± 1122 | 3.95 (2.02–6.02) | 55.3^d^ ± 26.3 |
| 3: 800 mg (n =3^a^) | 320 ± 125 | 5993 ± 2012 | 6197 ± 2217 | 3.85 (2.08–8.00) | 50.6^e^ |
| 4: 1200 mg (n = 1) | 336 | 5898 | 6175 | 7.93 | NC |
| **Osimertinib metabolite AZ5104** | | | | | |
| All: 200–1200 mg (n= 12^a^) | 43.9 ± 19.3 | 918 ±417 | 1.55 (0–8.17) | 36.2 ± 13.3 | 65.2^f^ ± 19.1 |
| **Osimertinib metabolite AZ7550** | | | | | |
| All: 200–1200 mg (n= 12^a^) | 42.2 ± 16.0 | 890 ± 346 | 7.98 (2.0–12.2) | 33.2 ± 12.2 | 47.3^f^ ± 7.40 |

AUC, area under the concentration-time curve; AUC_0–last_, AUC from time 0 until the last quantifiable time point; AUC_τ,_

AUC during a dosing interval; C_max_, maximum plasma concentration; NC, not calculable; SD, standard deviation; T_max_, time of maximum concentration; t_½,_ half-life.

^a^ Unless otherwise indicated.

^b^ Data from 11 patients were used to determine the pharmacokinetic parameter.

^c^ Data from 3 patients were used to determine the pharmacokinetic parameter.

^d^ Data from 2 patients were used to determine the pharmacokinetic parameter.

^e^ Data from 1 patient were used to determine the pharmacokinetic parameter.

^f^ Data from 4 patients were used to determine the pharmacokinetic parameter.

**Supplementary File 2. Biomarkers**

**Biomarkers methods**

Blood samples were collected to measure plasma levels of biomarkers that may correlate with response or toxicity to DS-1205c, including soluble AXL, interleukin (IL)-8, and osteopontin.

Tumor tissue samples from screening (fresh tumor biopsy or archived tumor specimen) were used to assess AXL expression in tumor tissue. IHC staining was performed, according to the manufacturer’s instructions, using a rabbit monoclonal antibody (C89E7) against AXL purchased from Cell Signaling Technology (Danvers, MA).

**Biomarkers and immunohistochemistry results**

Following the initial 1-week run-in period of DS-1205c monotherapy, plasma levels of soluble AXL increased by ≈1.6-fold from baseline. An increase of soluble AXL plasma level was shown in most patients and was not dose dependent. However, no significant changes of plasma IL-8 or osteopontin from baseline were observed in response to DS-1205c.

Before DS-1205c administration, 4 of the 11 evaluated tumor cell samples showed AXL-positive staining in ≥10% (range, 10–40%) of tumor cells. In normal nontumor cells in the stroma of the samples, 4 showed AXL-positive fibroblasts, and 10 showed AXL-positive immune cells.

In conclusion, treatment with DS-1205c increased plasma level of soluble AXL, a biomarker, but not the levels of IL-8 and osteopontin. The approximate 1.6-fold increase from baseline in soluble AXL levels was not dependent on the dosage of DS-1205c, suggesting that a plateau for this biomarker is achieved at low DS-1205c dosages. When the level of AXL expression in tumors was explored, AXL positivity was shown in 36% of 11 evaluable tumors before DS-1205c administration. This is similar to the results in patients treated with an EGFR TKI in a previous study, in which 47% (9 of 19) of evaluable tumors were AXL-positive after EGFR-TKI treatment [25]. Due to insufficient patient numbers, this study was unable to determine a clear correlation between these exploratory outcomes and clinical efficacy.

**Supplementary Data: Tables and Figures**

**Supplementary Table 1**. Summary of inclusion and exclusion criteria for study entry

| **Inclusion criteria** |  |
| --- | --- |
| Gender/age | Male or female aged ≥18 years |
| NSCLC related | Histologically or cytologically documented adenocarcinoma NSCLC |
|  | Locally advanced or metastatic NSCLC (not amenable to curative surgery or radiation) |
|  | Acquired resistance to an EGFR TKI (Jackman criteria); clinically defined as either a tumor with an *EGFR* mutation (e.g., *G719X*, exon 19 deletion, *L858R, L861Q*) associated with EGFR TKI sensitivity, or experience of clinical benefit from an EGFR TKI followed by systemic disease progression (RECIST v.1.1 or WHO) while receiving continuous EGFR TKI treatment |
|  | Demonstrated absence of the *EGFR T790M* mutation (*EGFR* mutation testing not required if previously treated with osimertinib) |
| Tumor related | ≥1 measurable lesion defined by RECIST v.1.1 |
|  | Able to provide archival tumor tissue from either a biopsy performed after progression during treatment with erlotinib, gefitinib, afatinib, or osimertinib, or ≥1 not previously irradiated lesion amenable to core biopsy and to undergo screening tumor biopsy |
| Treatment related | Currently receiving and able to discontinue erlotinib, gefitinib, or afatinib; or currently receiving osimertinib 80 mg/day and able to interrupt treatment |
|  | Continuous treatment with erlotinib, gefitinib, afatinib, or osimertinib for ≥6 weeks with well-controlled (grade <3) related toxicities at screening |
|  | Radiological documentation of disease progression while receiving continuous treatment with erlotinib, gefitinib, afatinib, or osimertinib |
| ECOG-PS | ECOG-PS of 0 or 1, with no deterioration over the previous 2 weeks |
| Bone marrow reserve and organ function | Adequate bone marrow reserve and organ function, defined as: platelet count ≥100 × 10^9^/L; hemoglobin ≥9.0 g/dL (transfusion and/or growth factor support allowed); ANC ≥1.5 🞨 10^9^/L; PT/aPTT ≤1.5 🞨ULN except patients on coumarin-derivative or similar anticoagulants, who had to have a PT-INR within the therapeutic range deemed appropriate by the investigator; serum creatinine ≤1.5 🞨 ULN, or Cockcroft-Gault CR_CL_ ≥50 mL/min (CR_CL_ required confirmation only if creatinine was >1.5 🞨 ULN); AST or ALT ≤3.0 🞨ULN without liver metastases or ≤5.0 🞨ULN with liver metastases; total bilirubin ≤1.5🞨 ULN without liver metastases or <3.0 🞨ULN with documented Gilbert’s Syndrome (unconjugated hyperbilirubinemia) or liver metastases; creatine kinase <1.5 🞨 ULN; lipase <2 🞨 ULN |
| Treatment washout before the first dose of DS-1205c | Undergo an adequate treatment washout period of ≥10 days for QTc prolonging medications and strong CYP3A4 s inducers, and ≥5 days for proton pump inhibitors (e.g., omeprazole, esomeprazole, lansoprazole, dexlansoprazole, pantoprazole) |
| Contraception | Females of reproductive/childbearing potential and males with a female sexual partner of child-bearing potential had to agree to use a highly effective form of contraception or avoid intercourse during and for ≥4 months after the last dose of DS-1205c |
| Other | Willing and able to: sign consent and other applicable forms; complete daily study mediation diary |
| **Exclusion criteria** |  |
| Histology | Evidence of small cell or small cell plus non-small cell histology in an original or screening tumor biopsy performed since progression |
| Mutations | Previous documentation of *ALK* fusion, *ROS1* fusion, *BRAF* V600E mutations, *RET* rearrangement, *HER2* mutation, or *MET* exon 14 skipping mutation (no new testing for these genomic alterations was required for screening) |
| Anticancer therapies before the first dose of DS-1205c | Treatment with any cytotoxic chemotherapy, investigational agent, or other anticancer drugs other than an EGFR TKI (within 14 days); immune checkpoint inhibitor therapy (within 30 days); major surgery, except vascular access procedures (within 4 weeks); radiotherapy to >30% of the bone marrow or with a wide field (within 4 weeks); palliative radiation therapy (within 2 weeks) |
| Other active malignancies | History of other active malignances within 3 years, except: adequately treated non-melanoma skin cancer; superficial bladder tumors (tumor stage ‘a’, ‘is’ or ‘1’); curatively treated *in situ* disease; or low-risk non-metastatic prostate cancer (Gleason score <7, following local treatment or undergoing active surveillance) |
| Conditions affecting drug absorption | Clinically significant malabsorption syndrome or other gastrointestinal disease (e.g., persistent diarrhea, or known sub-acute bowel obstruction NCI-CTCAE v5.0 grade ≥ 2, despite medical management) that could impact drug absorption |
| Retinal disease | Presence of retinal disease not due to neovascular age-related macular degeneration (e.g., significant diabetic retinopathy, glaucomatous retinal atrophy, retinal detachment) |
| Spinal cord compression and brain metastases | Spinal cord compression or clinically active brain metastases (defined as untreated and symptomatic, or required therapy with corticosteroids or anticonvulsants to control associated symptoms). Patients could be included in the study if they had: clinically inactive brain metastases; or treated brain metastases that were no longer symptomatic and did not require corticosteroids or anticonvulsants if patients had recovered from the acute toxic effect of radiotherapy. Before study enrollment, ≥2 weeks must have elapsed since the end of whole brain radiotherapy (≥1 week for stereotactic radiotherapy) |
| Related to QT interval prolongation | Mean QTc-F interval prolongation >450 ms (men) or >470 ms (women) in three successive measurements; any factors associated with increasing the risk of QTcF prolongation or arrhythmic events (e.g., congenital long QT syndrome, family history of long QT syndrome of unexplained sudden death at <40 years of age in 1^st^-degree relative); unable or willing to discontinue use of concomitant use of drugs known to prolong the QC interval |
| History of or current cardiac conditions | History of myocardial information; symptomatic NYHA Class II–IV congestive heart failure, unstable angina, or cardiac arrhythmia requiring antiarrhythmic treatment; LVEF <45% (ECHO or MUGA scan); any clinically important rhythm, conduction or morphology abnormalities of resting ECG (e.g., complete left bundle branch block, 2^nd^- or 3^rd^-degree heart block, PR interval >250 ms) |
| Interstitial lung disease | Any history of interstitial lung disease (pulmonary fibrosis or severe radiation pneumonitis) or was suspected to have such disease by imaging during screening |
| Other conditions (screening for chronic conditions was not required) | Any evidence of severe or uncontrolled systemic diseases (including uncontrolled hypertension), active bleeding diatheses or active infection (including HIV, or hepatitis B or C), psychiatric illness/social situations, substance abuse, or other undesirable factors for study participation or protocol compliance in the investigator's opinion |
| Pancreatitis | History of pancreatitis (past 6 months) |
| Pregnancy/lactation | Pregnancy (confirmed by a pregnancy test 7 days before enrolment) and lactating mothers (including women who were to temporarily interrupt breastfeeding) |

ALK, anaplastic lymphoma kinase; ALT, alanine aminotransferase; ANC, absolute neutrophil count; aPTT, activated partial thromboplastin time; AST, aspartate aminotransferase; CR_Cl_, creatinine clearance; CYP, cytochrome P450; ECG, electrocardiogram; ECHO, echocardiogram; ECOG-PS, Eastern Cooperative Oncology Group-performance status; EGFR, epidermal growth factor receptor; HER2, human epidermal growth factor receptor 2; HIV, human immunodeficiency virus; INR, International Normalized Ratio; LVEF, left ventricular ejection fraction; MUGA, multigated acquisition; NCI-CTCAE v5.0, National Cancer Institute-Common Terminology Criteria for Adverse Events version 5.0; NSCLC, non-small cell lung cancer; NYHA, New York Heart Association; PT, prothrombin time; QTcF ,QT interval corrected for heart rate using Fridericia’s formula; RECIST v.1.1, Response Evaluation Criteria in Solid Tumors version 1.1; RET, rearranged during transfection; ROS1, ROS proto-oncogene 1; TKI, tyrosine kinase inhibitor; ULN, the upper limit of normal; WHO, World Health Organization.

**Supplementary Table 2** Baseline patient demographics and characteristics, *EGFR* mutations and prior EGFR-TKI treatment (full analysis set)

| **Parameter** | **Cohort: DS-1205c twice-daily dosage** | | | | |
| --- | --- | --- | --- | --- | --- |
|  | All: 200–1200 mg  (n = 13) | 1: 200 mg  (n = 6) | 2: 400 mg  (n = 3) | 3: 800 mg  (n = 3) | 4: 1200 mg  (n = 1) |
| **Demographic/characteristic** | | | | | |
| Age (years), median (range) | 64.0 (45–88­) | 57.5 (45–77) | 73.0 (61–88) | 64.0 (63–68) | 70.0 |
| Sex, n | 3 M; 10 F | 6 F | 1 M; 2 F | 2 M; 1 F | M |
| ECOG-PS, n | 7 PS=0; 6 PS=1 | 2 PS=0; 4 PS=1 | 1 PS=0; 2 PS=1 | 3 PS=0 | PS=1 |
| Weight (kg), median, (range) | 52.5 (42.8–74.6) | 50.75 (42.8–74.6) | 53.8 (50.3–56.1) | 60.9 (43.5–61.4) | 51.0 |
| ***EGFR* mutation status** | | | | | |
| *G719A/S/C*, n | 4 Neg; 9 NK | 2 Neg; 4 NK | 3 NK | 1 Neg; 2 NK | Neg |
| *T790M*, n | 4 Neg; 2 Pos; 7 NK | 2 Neg; 1 Pos; 3 NK | 1 Pos; 2 NK | 1 Neg; 2 NK | Neg |
| *L858R*, n | 3 Neg; 2 Pos; 8 NK | 2 Neg; 4 NK | 3 NK | 2 Pos; 1 NK | Neg |
| Exon 19 deletion, n | 1 Neg; 4 Pos; 8 NK | 3 Pos; 3 NK | 3 NK | 1 Neg; 2 NK | Pos |
| *S768I*, n | 4 Neg; 9 NK | 2 Neg; 4 NK | 3 NK | 1 Neg; 2 NK | Neg |
| Exon 20 insertion, n | 4 Neg; 9 NK | 2 Neg; 4 NK | 3 NK | 1 Neg; 2 NK | Neg |
| *L861Q*, n | 4 Neg; 9 NK | 2 Neg; 4 NK | 3 NK | 1 Neg; 2 NK | Neg |
| **Prior cancer medical therapy (patients could list ≥ 1 therapy)** | | | | | |
| Any, n | 13 | 6 | 3 | 3 | 1 |
| Afatinib, n | 5 | 3 | 1 | 1 |  |
| Erlotinib, n | 6 | 2 | 1 | 2 | 1 |
| Gefitinib, n | 5 | 2 | 1 | 2 |  |
| Osimertinib, n | 8 | 4 | 3 | 1 |  |
| Other, n | 9 | 4 | 2 | 2 | 1 |
| **EGFR-TKI treatment immediately prior to DS-1205c** | | | | | |
| Any, n | 13 | 6 | 3 | 3 | 1 |
| Afatinib, n | 2 | 2 |  |  |  |
| Erlotinib, n | 3 |  |  | 2 | 1 |
| Osimertinib, n | 8 | 4 | 3 | 1 |  |

ECOG-PS, Eastern Cooperative Oncology Group performance status; EGFR, epidermal growth factor receptor; F, female; M, male; Neg, negative, NK, not known; Pos, positive; PS=0, ECOG PS = 0; PS=1, ECOG PS = 1; TKI, tyrosine kinase inhibitor.

**Supplementary Fig. 1** Kaplan-Meier plot of (a) progression-free survival; (b) overall survival (full analysis set)


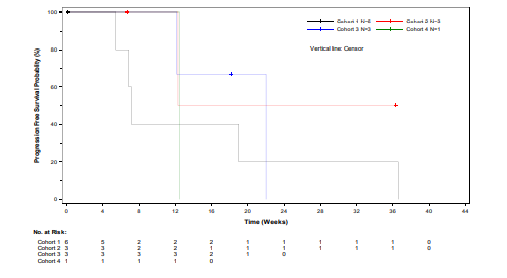

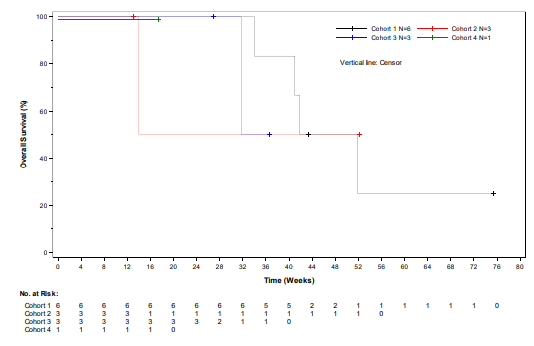


(b)

(a)
